# Analytical and Clinical Comparison of Two Fully Automated Immunoassay Systems for the Diagnosis of Celiac Disease

**DOI:** 10.1155/2014/371263

**Published:** 2014-03-13

**Authors:** Gabriella Lakos, Gary L. Norman, Michael Mahler, Peter Martis, Chelsea Bentow, Debby Santora, Alessio Fasano

**Affiliations:** ^1^INOVA Diagnostics, Inc., 9900 Old Grove Road, San Diego, CA 92131-1638, USA; ^2^University of Maryland, Baltimore, MD 21201, USA; ^3^Center for Celiac Research, Massachusetts Hospital for Children, Boston, MA 02114, USA

## Abstract

*Objective.* Here we compared analytical and clinical performance characteristics of two novel automated assay systems for the detection of celiac disease (CD) specific antibodies: QUANTA Flash (INOVA Diagnostics, Inc.) and EliA (Thermo Scientific). * Methods.* A total of 74 biopsy-proven CD patients (2 with IgA deficiency) and 138 controls were tested by both methods. * Results. *Sensitivities of QUANTA Flash assays ranged from 35.1% to 90.5% and specificities from 96.4% to 99.3%, while sensitivities for EliA assays ranged from 37.8% to 90.5% (equivocal considered positive) and specificities from 97.1% to 100.0%. Good qualitative agreement was found between all assays. Thirty-four (50.0%) of the 68 QUANTA Flash h-tTG IgA positive results were higher than 10 times the upper limit of normal (ULN). In contrast, only 22.8% of the EliA tTG IgA positive samples were >10x ULN. Seventy-three (98.6%) biopsy-proven CD patients were correctly identified with the QUANTA Flash h-tTG IgA+DGP IgG combination, while 64 (86.5%) and 72 (97.3%) (depending on equivocal range) were identified with the same combination of EliA assays. * Conclusion.* The QUANTA Flash CD assays have outstanding clinical performance. Of particular clinical significance, in light of proposals to decrease the absolute necessity of biopsy, was the demonstration that 50% of the QUANTA Flash h-tTG IgA results were >10x ULN.

## 1. Introduction

Celiac disease (CD) is characterized by a life-long intolerance to gluten from wheat, barley, or rye. Screening studies have shown that it is a very common disease affecting about 1% of the western population and there is increasing recognition that it is present and perhaps increasing in nontraditional areas such as the Middle East, North Africa, and India [1–4]. Although CD mostly affects the gastrointestinal tract, extraintestinal manifestations defined as nonclassical CD, including anemia, bone disease, infertility, unfavorable pregnancy outcome, lymphoma, and liver disease occur in a subpopulation of patients [5]. Consequently, CD can be considered a systemic autoimmune disease with treatment involving a gluten-free diet; however, recent research has explored novel therapies and other dietary factors [6, 7]. The diagnosis of CD typically consists of three parts: serology, small bowel biopsy, and remission of the disease following adherence to a gluten-free diet. The serological tests for CD include assays to detect antibodies to human tissue transglutaminase (tTG), deamidated gliadin peptide (DGP, detection of antibodies to whole gliadin is not appropriate for CD diagnosis), or endomysium and are frequently followed by intestinal biopsy if positive [4, 8]. While the gold standard for the unequivocal diagnosis of CD is the demonstration of villous blunting on duodenal biopsy, increasing attention has been focused on whether serological assays could be used to significantly decrease the need for biopsy [9–11].

In 2012 the European Society for Pediatric Gastroenterology, Hepatology and Nutrition (ESPGHAN) published new guidelines which included the proposal that pediatric patients with anti-tTG IgA antibodies ≥10x the upper limit of normal (ULN) for curve-based assays, together with gluten-dependent symptoms and the presence of HLA DQ2 and/or DQ8, may consider omission of duodenal biopsy [12]. Clinical response to gluten withdrawal and decline of antibody is considered the confirmation of the diagnosis. The QUANTA Flash h-tTG IgA and IgG, and the QUANTA Flash DGP IgA and IgG are new, fully automated, microparticle chemiluminescent immunoassays (CIA). Our goal in this study was to assess and compare some of the analytical and clinical performance characteristics of the new automated CIA (FEIA) system with a fluoroenzyme immunoassay automated assay system for the diagnosis of CD, as well as assessing, in adult patients with celiac disease, the frequency values meeting the 10 times ULN by the CIA and FEIA methodologies.

## 2. Materials and Methods

### 2.1. Sera

A total of 229 patient samples were tested in the study. After excluding CD patients on gluten-free diet and samples with insufficient quantity to run all tests, the cohort included 74 biopsy-proven adult CD patients (2 of them with selective IgA deficiency) and 138 controls, including age and sex matched healthy controls (*n* = 129), as well as patients with food allergy (*n* = 3), inflammatory bowel disease (*n* = 3), and rheumatoid arthritis (*n* = 3). Since the study focused on adult patients with CD, the ages for CD patients ranged from 19 to 83, with a median age of 48 (SD = 15.52). The control group ages ranged from 7 to 89, with a median age of 47 (SD = 16.62). Although most controls were adult, only 6 controls were pediatric (three age 7, two age 9, and one age 11). Out of the 74 CD patients, sixty were female and fourteen were male, while the controls had 101 females and 37 males. In terms of ethnicity, the entire sample population (*n* = 212) was also mostly Caucasian (*n* = 201), but there were four Hispanic, two Armenian, and five with no given information. Samples were collected at the University of Maryland Center for Celiac Research. The study was approved by the University of Maryland Institutional Review Board. Patient identity was not disclosed and the data was anonymously used in accordance with the latest version of the Helsinki Declaration of human research ethics.

### 2.2. QUANTA Flash Assays

The QUANTA Flash h-tTG IgA and IgG, and DGP IgA and IgG assays (INOVA Diagnostics, Inc., San Diego, CA, USA) are used on the BIO-FLASH instrument (Biokit s.a., Barcelona, Spain), a fully automated chemiluminescent immunoanalyzer. The principle of the BIO-FLASH system has recently been described [13]. The QUANTA Flash assays utilize recombinant human tTG antigen and synthetic DGP peptides coated onto paramagnetic beads. Bound antibodies are detected with isoluminol-conjugated anti-human IgA and IgG secondary antibodies, and the signal is measured as Relative Light Units (RLUs) by the BIO-FLASH optical system. The RLUs are proportional to the amount of isoluminol conjugate that is bound to the human IgA or G, which in turn is proportional to the amount of autoantibodies bound to the antigen on the beads. For all of the QUANTA Flash assays used in this study, ≥20 chemiluminescent units (CU) are considered positive and none of the assays have an equivocal range. The analytical measuring range (AMR) for each of the QUANTA Flash assays can be found in Table 1. Additionally, to aid in the measurement of samples that run above the AMR, the BIO-FLASH software has an Auto-Rerun option available. If this option is selected, the instrument will automatically rerun any sample that has a result above the AMR by further diluting it by a factor of 10 and calculating the actual CU using this additional dilution factor.

### 2.3. FEIA Assays

FEIA assays used in this study are the EliA Celikey IgA, and IgG as well as the EliA Gliadin IgA and IgG (Thermo Scientific, Phadia GmbH, Freiburg, Germany). Single well-based, automated fluoroenzyme immunoassays (FEIAs) were performed using a fully automated microplate system. The AMR for each of the FEIAs can be found in Table 1. For all of the FEIAs used in this study, >10 Units/mL is considered positive. The assays have an equivocal range defined between 7 and 10 Units/mL. For statistical purposes, equivocal results were considered either negative or positive in the analysis, as indicated in the text. The assays were performed on the automated Phadia 250 (Freiburg, Germany) instrument according to the manufacturer's instructions.

### 2.4. Duodenal Biopsy

All CD patients included in this study were biopsy proven. The duodenal biopsies were performed during routine diagnostic endoscopy procedures. Forty-five biopsies were obtained from bulb and duodenal mucosa, immediately fixed and then processed for histological analysis. Due to ethics regulations, no biopsies were performed on the disease controls included in this study.

### 2.5. EMA

Anti-endomysial antibody (EMA) IgA test was performed on all biopsy-proven CD patients (*n* = 74) as well as a significant amount of the disease controls included in this study (*n* = 102 out of 138 controls). Thirty-six healthy controls did not have anti-EMA IgA testing. EMA was detected by indirect immunofluorescence assays (Scimedx, Denville, NJ, USA) using monkey esophagus as substrate and as recommended by the manufacturer. Values above 1 : 10 were considered positive.

### 2.6. Statistical Analyses

The data were statistically evaluated using the Analyse-it software (Version 1.62; Analyse-it Software, Ltd., Leeds, UK). Diagnostic sensitivity and specificity of all tests were calculated and compared. Diagnostic efficacy was assessed by receiver operating characteristics (ROC) analysis. The number (percentage) of samples that fell within the AMR of the tTG IgA test was determined, together with the number (percentage) of samples that were ≥10 times the cutoff of the tTG IgA assay. Spearman's correlation and Cohen's* kappa* agreement test were carried out to analyze the agreement between portions. Cluster analysis was used to illustrate the relationship between different assays and to display the reactivity pattern of the patients [14]. Hierarchical clustering was performed using average linkage clustering where patient correlation was performed centered and the reactivities uncentered.

## 3. Results

### 3.1. Clinical Sensitivity and Specificity

**Figure 1:**
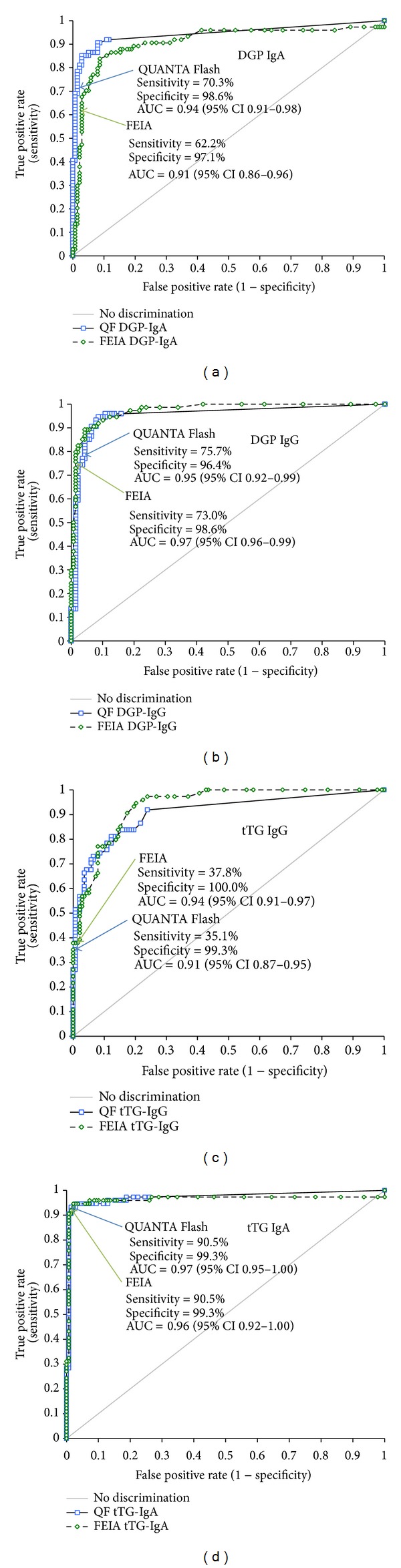
Comparative ROC curve analyses. ROC for QUANTA Flash DGP IgA and FEIA DGP IgA is shown in (a), for QUANTA Flash (QF) DGP IgG and FEIA DGP IgG in (b), for QUANTA Flash h-tTG IgG and FEIA IgG in (c), and for QUANTA Flash h-tTG IgA and FEIA IgA in (d). The ROC curves were similar for tTG IgA, tTG IgG, and DGP IgG. For DGP IgA, the AUC (especially in the clinically relevant area) was higher for QF versus FEIA. Note: clinical sensitivity and specificity as well as arrows pointing to cutoffs in this figure are for equivocal samples considered as positive for FEIA assays.

**Figure 2:**
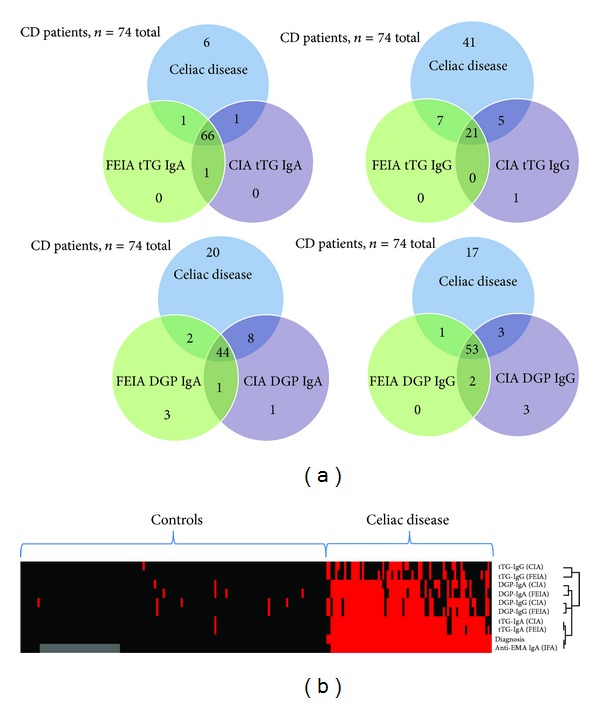
(a) Venn diagram depiction of clinical sensitivity and specificity as well as assay agreement for tTG IgA/IgG and DGP IgA/IgG assays. Upper circle in each group represents all 74 patients with celiac disease and shows presence/overlap of markers (b) Cluster analysis. The cluster analysis shows that the majority of celiac disease patients have multiple positive results. Some of the controls also show positive results by different methods. Isolated vertical lines indicate specimens positive for multiple markers and potentially at increased risk for celiac disease. Red: positive; black: negative; grey: no data available.

**Figure 3:**
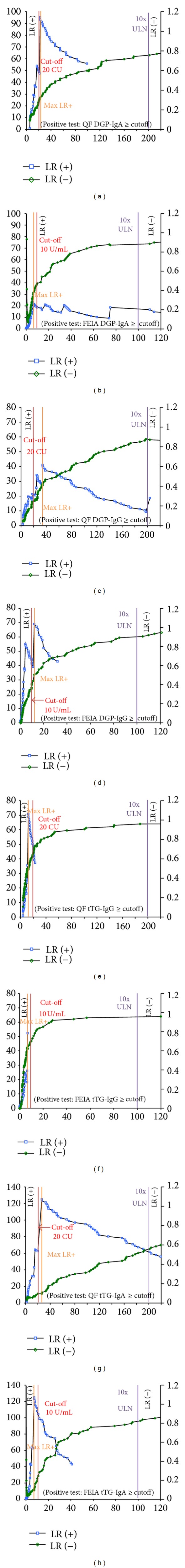
Likelihood ratio plots for all assays. The positive and negative likelihood ratios (*y*-axis) are plotted against the titer of the antibodies (*x*-axis). Likelihood ratios are shown at the cutoff (red line) and at the highest positive likelihood (orange line). NOTE: likelihood ratios are illustrated based on values depicted by the software and displayed in a graph. Likelihood ratios between two data points might be different from the real values. QF = QUANTA Flash; FEIA = fluoroenzyme immunoassay.

Clinical sensitivities and specificities were calculated for all assays. The highest sensitivity for biopsy-proven CD patients was found for the QUANTA Flash h-tTG IgA (90.5%). The FEIA counterpart had 75.7% sensitivity when equivocal results were considered negative, but equal to the CIA (90.5%) when those were considered positive. Both the QUANTA Flash DGP IgA and IgG assays had higher sensitivity (70.3% and 75.7%, resp.) compared to the FEIA DGP IgA and IgG tests, regardless of the categorization of the equivocal results (Table 2). Specificity was high for all assays, ranging from 96.4% for QUANTA Flash DGP IgG to 100% for FEIA tTG IgG (Table 2). ROC curve analysis was performed for all assays showing AUC values ranging from 0.91 to 0.98 (QUANTA Flash) and from 0.91 to 0.97 (FEIA) (Figure 1). Sensitivities, specificities, and agreements between methods are also depicted as Venn diagrams in Figure 2(a). Likelihood plots were generated to analyze the LRs as a function of the antibody titer. LR plots were similar between both assays systems for tTG IgA and tTG IgG, but different for DGP IgA and DGP IgG (Figure 3). When analyzing the combined sensitivity of the celiac assays, it was determined that 73 (98.6%) out of all biopsy-proven CD patients were correctly identified with the QUANTA Flash h-tTG IgA + DGP IgG combination, while 64 (86.5%) and 72 (97.3%) (depending on how equivocal results are considered) were identified with the same combination of FEIA assays.

### 3.2. Cluster Analysis

To illustrate the reactivity of various assays in relation to the diagnosis, we performed a cluster analysis. The cluster analysis shows that the majority of CD patients have multiple positive results (Figure 2(b)). Some of the controls also show positive results by different methods. Both tTG IgA assays cluster closest to the diagnosis of CD. The assays with the biggest distance to the diagnosis are the two tTG IgG assays. Additionally, the cluster analysis included the anti-EMA IgA results, which also shows that anti-EMA IgA and both tTG IgA assays cluster closest to the diagnosis of CD.

### 3.3. EMA Results

Seventy-two out of 74 (97.3%) biopsy-proven CD patients tested positive by anti-EMA IgA test. The two CD patients which did not test positive for anti-EMA IgA were IgA deficient. Additionally, all disease controls tested by anti-EMA IgA (*n* = 102) were negative.

### 3.4. Qualitative Agreements between Assays

**Figure 4:**
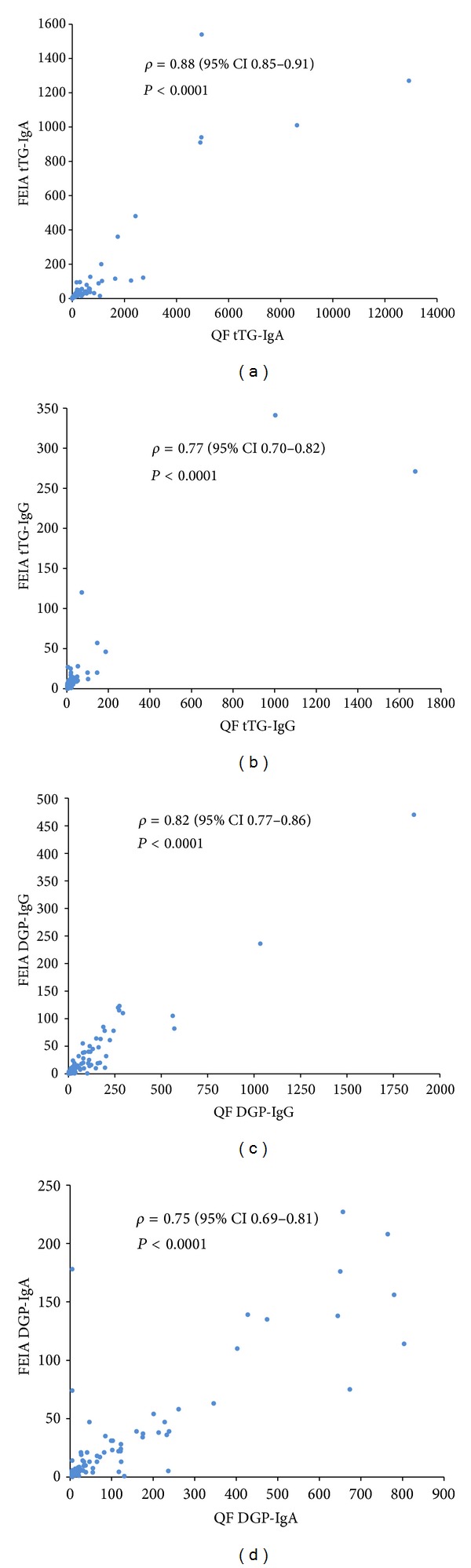
Spearman's correlation (celiac disease patients and controls, *n* = 212) among different assays: (a) tTG-IgA, (b) tTG-IgG, (c) DGP-IgG, and (d) DGP-IgA; QF = QUANTA Flash; FEIA = fluoroenzyme immunoassay.

Good qualitative agreements were found between the results of the CIA and FEIAs, with the highest total percent agreement of 99.1% (*kappa* = 0.98) between the tTG IgA assays (when FEIA equivocal results were considered positive), and the lowest was a total agreement of 92.0% (*kappa* = 0.77) between the DGP IgA and IgG assays. Percent agreement and Cohen's* kappa *values between the QUANTA Flash (QF) assays and FEIAs can be found in Table 3. Additionally, good quantitative correlation was found between unit values obtained with the QUANTA Flash assays and FEIAs, with Spearman's rho ranging from 0.75 for DGP IgA to 0.88 for tTG IgA (see Figure 4).

### 3.5. Upper Limit of Normal and the Analytical Measuring Range

**Figure 5:**
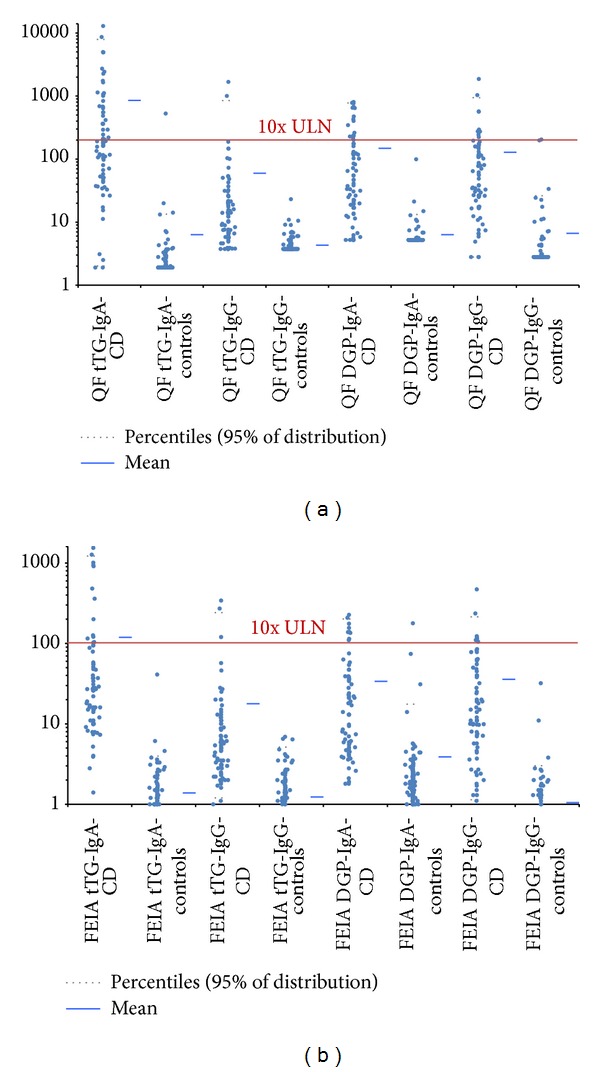
Comparative descriptive analysis. Antibody levels for QUANTA Flash (QF) assays in (a) and fluoroenzyme immunoassays (FEIAs) in (b). The 10 upper limit of normal is indicated by the red line.

According to the newly published ESPGHAN guidelines, omission of duodenal biopsy may be considered for pediatric patients with anti-tTG IgA antibodies ≥10x the upper limit of normal (ULN) for curve-based assays together with gluten-dependent symptoms and the presence of HLA DQ2 and/or DQ8. This guideline was used to analyze both tTG IgA assays in this study with an adult population of CD patients. Thirty-four (50.0%) out of the 68 tTG IgA positive results with the QUANTA Flash assay were higher than 10 times the ULN. To illustrate the antibody levels measured on the QUANTA Flash assays and FEIA, comparative descriptive analysis was performed on both CD patients and disease controls (Figure 5). Only 13 out of the 57 positive FEIA tTG IgA results (22.8%) were higher than 10 times the ULN. We also examined the number of patients whose tTG IgA results fell outside the AMR for the assay. Eight tTG IgA results were above the AMR with FEIA and three with the QUANTA Flash assays.

## 4. Discussion

Serology is an important part in the identification and diagnosis of CD and it has been suggested that, in cases of high titers of tTG IgA (>10 ULN), biopsy might be omitted [12]. It is essential, therefore, that highly reliable and accurate assays are utilized to detect the CD-specific antibodies from both a diagnostic and follow-up point of view. In the present study we evaluated anti-tTG (IgG, IgA) and anti-DGP (IgG, IgA) antibody assays on two fully automated systems to assess their accuracy and performance. Both assay systems showed similar AUC values by ROC analyses. Clinical sensitivities were comparable (except for DGP IgA) when equivocal FEIA results were considered positive but were lower for the FEIA when equivocal results were considered negative. These results imply that performance differences mainly originate from different cutoffs.

Seventy-three (98.6%) out of all biopsy-proven CD patients were correctly identified with the QUANTA Flash h-tTG IgA + DGP IgG combination, while 64 (86.5%) and 72 (97.3%) (depending on how equivocal results are considered) were identified with the same combination of FEIA. Although the number of patients that tested positive by QUANTA Flash DGP IgG but negative by tTG IgA was small in this study (8.1%, *n* = 6 patients), additional testing with the DGP assays and tTG IgG is still desirable alongside the tTG IgA test to aid in the diagnosis of CD. In circumstances where the patient is IgA deficient or has a low positive result for tTG IgA, multiple positivity by the other tests adds clinical confidence for a life-long diagnosis [15]. We also confirmed the increasing positive likelihood ratio for CD with increasing titers of CD specific antibodies which has been demonstrated in previous studies [16]. More positive results fell within the AMR of the QUANTA Flash h-tTG IgA assay than that of the FEIA counterpart. Eight tTG IgA results were above the AMR of the FEIA and three with the QUANTA Flash assays. Autorerun of samples with results above the AMR is automatic with the QUANTA Flash assays, but manual dilution and a second run are required with the FEIA for accurate quantitation. The broad AMR is especially beneficial since the QUANTA Flash h-tTG was shown to be useful in monitoring of disease activity in a pediatric population of CD patients [17]. Thirty-four (50.0%) of the 68 QUANTA Flash h-tTG IgA positive results were higher than 10 times the ULN. In contrast, only 13 of the 57 positive FEIA tTG IgA results (22.8%) were higher than 10 times the ULN. The new ESPGHAN guidelines (published in 2012) suggest this antibody level as a threshold for selecting pediatric patients who can potentially avoid duodenal biopsy, and whose disease may be diagnosed based solely on laboratory assays [12]. We have shown here, for the first time, that the 10 times ULN ESPGHAN guideline can also be applied to adult patients. More positive results for the QUANTA Flash assay were ≥10 times the cutoff, thereby identifying patients who could potentially avoid duodenal biopsy according to the ESPGHAN guidelines. This could translate to a significant decrease in the need for duodenal biopsy with its associated costs and inconvenience for the patient [6, 7].

## 5. Conclusion

Our data demonstrate that QUANTA Flash h-tTG IgA is a reliable test for the diagnosis of CD with rapid turnaround time (30 minutes). QUANTA Flash h-tTG IgG, DGP IgA, and DGP IgA show similar performance characteristics to FEIA for the detection of celiac specific antibodies. The broad AMR of the QUANTA Flash h-tTG IgA assay results in more than twice as many biopsy-proven celiac patients meeting the 10 ULN criteria as a similar FEIA and thus possibly significantly reducing the need for duodenal biopsy for the diagnosis of CD.

## Figures and Tables

**Table 1:** Analytical measuring range for celiac disease assays.

Assay	h-tTG IgA	h-tTG IgG	DGP IgA	DGP IgG
chemiluminescent immunoassay	1.9–4965.5 CU	3.75–2560.0 CU	5.2–2367.3 CU	2.8–1936.7 CU
fluoroenzyme immunoassay	0.1–128 U/mL	0.5–600 U/mL	0.2–213 U/mL	0.2–192 U/mL

**Table 2:** Clinical sensitivity and specificity.

Assay	Sensitivity % (95% CI)	Specificity % (95% CI)	LR+/LR−
QF h-tTG IgA	90.5 (81.5–96.1)	99.3 (96.0–100.0)	124.95/0.10
h-tTG IgA FEIA (equivocal = positive)	90.5 (81.5–96.1)	99.3 (96.0–100.0)	124.95/0.10
h-tTG IgA FEIA (equivocal = negative)	75.7 (64.3–84.9)	99.3 (96.0–100.0)	104.43/0.25
QF h-tTG IgG	35.1 (24.4–47.1)	99.3 (96.0–100.0)	48.49/0.65
h-tTG IgG FEIA (equivocal = positive)	37.8 (26.8–49.9)	100.0 (97.4–100.0)	+∞/0.62
h-tTG IgG FEIA (equivocal = negative)	27.0 (17.4–38.6)	100.0 (97.4–100.0)	+∞/0.73
QF DGP IgA	70.3 (58.5–80.3)	98.6 (94.9–99.8)	48.49/0.30
DGP IgA FEIA (equivocal = positive)	62.2 (50.1–73.2)	97.1 (92.7–99.2)	21.45/0.39
DGP IgA FEIA (equivocal = negative)	52.7 (40.7–64.4)	97.1 (92.7–99.2)	18.18/0.49
QF DGP IgG	75.7 (64.3–84.9)	96.4 (91.7–98.8)	20.89/0.25
DGP IgG FEIA (equivocal = positive)	73.0 (61.4–82.6)	98.6 (94.9–99.8)	50.35/0.27
DGP IgG FEIA (equivocal = negative)	56.8 (44.7–68.2)	98.6 (94.9–99.8)	39.16/0.44

**Table 3:** Qualitative agreements between all assays.

Assay	% PPA (95% CI)	% NPA (95% CI)	% TPA (95% CI)	*kappa* (95% CI)
QF versus FEIA tTG IgA (FEIA equiv = positive)	98.5 (92.1–100.0)	99.3 (96.2–100.0)	99.1 (96.6–99.9)	0.98 (0.95–1.01)
QF versus FEIA tTG IgA (FEIA equiv = negative)	100.0 (93.7–100.0)	92.9 (87.7–96.4)	94.8 (90.9–97.4)	0.88 (0.80–0.95)
QF versus FEIA tTG IgG (FEIA equiv = positive)	75.0 (55.1–89.3)	96.7 (93.0–98.8)	93.9 (89.7–96.7)	0.73 (0.59–0.87)
QF versus FEIA tTG IgG (FEIA equiv = negative)	80.0 (56.5–94.3)	94.3 (90.0–97.1)	92.9 (88.6–96.0)	0.64 (0.48–0.81)
QF versus FEIA DGP IgA (FEIA equiv = positive)	93.0 (80.9–98.5)	91.7 (86.5–95.4)	92.0 (87.5–95.3)	0.77 (0.67–0.88)
QF versus FEIA DGP IgA (FEIA equiv = negative)	90.0 (78.2–96.7)	94.4 (89.7–97.4)	93.4 (89.2–96.3)	0.82 (0.73–0.91)
QF versus FEIA DGP IgG (FEIA equiv = positive)	98.2 (90.4–100.0)	96.2 (91.8–98.6)	96.7 (93.3–98.7)	0.92 (0.86–0.98)
QF versus FEIA DGP IgG (FEIA equiv = negative)	100.0 (92.0–100.0)	89.9 (84.3–94.0)	92.0 (87.5–95.3)	0.79 (0.69–0.88)

Note: PPA: positive percent agreement, NPA: negative percent agreement, TPA: total percent agreement, QF: QUANTA Flash; FEIA: fluoroenzyme immunoassay.

## Abbreviations

AMR: Analytical measuring range
AUC: Area under the curve
CD: Celiac disease
CIA: Chemiluminescent immunoassay
ESPGHAN: European Society for Pediatric Gastroenterology, Hepatology and Nutrition
FEIA: Fluoroenzyme immunoassay
ROC: Receiver-operating characteristics
ULN: Upper limit of normal.
